# Supplementary material for: Association between gonadal hormones and osteoporosis in schizophrenia patients undergoing risperidone monotherapy: a cross-sectional study
Source: PeerJ. 2021 Apr 27;9:e11332. doi: 10.7717/peerj.11332 (PMC8086585; doi:10.7717/peerj.11332)
Supplement: Supplemental Information 2 [file peerj-09-11332-s002.docx]

| Number | Name | Type | Values |  |
| --- | --- | --- | --- | --- |
| 1 | Sex | Categorical variable | 1(male),2(female) | Nominal |
| 2 | Age | Continuous variable | - | Scale |
| 3 | Drinking | Categorical variable | 0(no),1(yes) | Nominal |
| 4 | Smoking | Categorical variable | 0(no),1(yes) | Nominal |
| 5 | BMD (bone mineral density) | Continuous variable | - | Scale |
| 6 | Osteoporosis | Categorical variable | 0(no),1(yes) | Nominal |
| 7 | Estradiol | Continuous variable | - | Scale |
| 8 | Progesterone | Continuous variable | - | Scale |
| 9 | Height | Continuous variable | - | Scale |
| 10 | Weight | Continuous variable | - | Scale |
| 11 | Total disease courses | Continuous variable | - | Scale |
| 12 | The daily dosage of risperidone | Continuous variable | - | Scale |
| 13 | Testosterone | Continuous variable | - | Scale |
| 14 | Prolactin | Continuous variable | - | Scale |
| 15 | FSH | Continuous variable | - | Scale |
| 16 | LH | Continuous variable | - | Scale |
| 17 | Participants | Categorical variable | 0(controls),1(patients) | Nominal |
